# Insights Gained From Zebrafish Models for the Ciliopathy Joubert Syndrome

**DOI:** 10.3389/fgene.2022.939527

**Published:** 2022-06-30

**Authors:** Tamara D. S. Rusterholz, Claudia Hofmann, Ruxandra Bachmann-Gagescu

**Affiliations:** ^1^ Institute of Medical Genetics, University of Zurich, Schlieren, Switzerland; ^2^ Department of Molecular Life Sciences, University of Zurich, Zürich, Switzerland

**Keywords:** cilia, ciliopathies, Joubert syndrome, zebrafish, retina, CRISPR/Cas9, morpholino (MO)

## Abstract

Cilia are quasi-ubiquitous microtubule-based sensory organelles, which play vital roles in signal transduction during development and cell homeostasis. Dysfunction of cilia leads to a group of Mendelian disorders called ciliopathies, divided into different diagnoses according to clinical phenotype constellation and genetic causes. Joubert syndrome (JBTS) is a prototypical ciliopathy defined by a diagnostic cerebellar and brain stem malformation termed the “Molar Tooth Sign” (MTS), in addition to which patients display variable combinations of typical ciliopathy phenotypes such as retinal dystrophy, fibrocystic renal disease, polydactyly or skeletal dystrophy. Like most ciliopathies, JBTS is genetically highly heterogeneous with ∼40 associated genes. Zebrafish are widely used to model ciliopathies given the high conservation of ciliary genes and the variety of specialized cilia types similar to humans. In this review, we compare different existing JBTS zebrafish models with each other and describe their contributions to our understanding of JBTS pathomechanism. We find that retinal dystrophy, which is the most investigated ciliopathy phenotype in zebrafish ciliopathy models, is caused by distinct mechanisms according to the affected gene. Beyond this, differences in phenotypes in other organs observed between different JBTS-mutant models suggest tissue-specific roles for proteins implicated in JBTS. Unfortunately, the lack of systematic assessment of ciliopathy phenotypes in the mutants described in the literature currently limits the conclusions that can be drawn from these comparisons. In the future, the numerous existing JBTS zebrafish models represent a valuable resource that can be leveraged in order to gain further insights into ciliary function, pathomechanisms underlying ciliopathy phenotypes and to develop treatment strategies using small molecules.

## Introduction

Ciliopathies are a group of human Mendelian disorders caused by dysfunction of primary cilia. Cilia are evolutionarily conserved hair-like organelles that protrude from virtually every vertebrate cell. The basic structure of a primary cilium consists of nine microtubule doublets (A- and B-tubules) arranged in a circular array. This microtubule scaffold is ensheathed by a membrane harboring specific components important for signal transduction (Emmer et al., 2010; Rohatgi and Snell, 2010), which is the main function of this organelle. Primary cilia are structurally divided into three compartments: 1) The basal body (BB) is derived from the centriole apparatus and is the starting point of cilium formation (Keeling et al., 2016). 2) The transition zone (TZ) is apical to the basal body and has a gatekeeper function for the cilium, controlling entry and exit of ciliary proteins (Szymanska and Johnson, 2012). 3) The axoneme protrudes from the basal body and defines the shape of the primary cilium (Singla and Reiter, 2006; Goetz and Anderson, 2010).

While the main role of primary cilia is signal transduction, these organelles have adopted diverse tissue-specific biological functions. Sensory signals for example are perceived in specialized cilia: In the retina, the outer segment of photoreceptors is a highly modified cilium which is required for light sensation (Kennedy and Malicki, 2009; Bachmann-Gagescu and Neuhauss, 2019), while olfaction is transduced by primary cilia on olfactory neurons (Singla and Reiter, 2006). Beyond such specialized sensory signals, primary cilia are equally important for sensing the environment and for transducing signals for crucial developmental pathways such as Hedgehog (Hh) signaling (Ishikawa and Marshall, 2011). Components of this pathway, which control key aspects of development such as patterning of the central nervous system (CNS) or limb buds, proliferation and differentiation of multiple cell types, localize to the cilium in a Hh-ligand dependent manner (Bangs and Anderson, 2017). Other developmental signaling pathways such as Wnt are also associated with primary cilia function, whereby the precise link is less well defined than for Hh signaling (Wheway et al., 2018).

To ensure their signaling function, primary cilia exert a tight control on the protein and lipid composition of their membrane, in order to concentrate receptors and channels required to perceive and regulate external stimuli. Since primary cilia lack protein translation machinery, all ciliary components need to be transported from the cytosol into the primary cilium, which is achieved through a combination of controlled vesicle trafficking and the conserved intraflagellar transport (IFT) system (Nachury et al., 2010).

In contrast to the single primary non-motile cilium present on most cells, motile cilia are usually present in multiple copies on one cell and beat in a synchronized manner to generate directed fluid flow. In humans, motile cilia are found only on a few specialized cell types such as the ependymal cells lining the ventricular surface in the brain, epithelial cells in the airways and in the reproductive tract (Goetz and Anderson, 2010). Different to primary cilia, motile cilia have an additional central microtubule pair, as well as dynein arms on the A-tubule to enable movement (Mitchell, 2007; Yamaguchi et al., 2018). Intermediates between primary and motile cilia are the motile cilia on cells of the embryonic node, important for left/right organization, which are single on a given cell and which lack the central microtubule pair (Babu and Roy, 2013).

Dysfunction of cilia leads to a group of disorders called ciliopathies. Dysfunction of cilium motility causes primary ciliary dyskinesia (PCD), which affects only organs where motile cilia are present, leading to recurrent respiratory infections, laterality defects and infertility. The larger group of “primary ciliopathies” includes disorders which can affect almost every organ system (Mitchison and Valente, 2017). Typical phenotypes include CNS malformations or dysfunction, retinal degeneration, liver fibrosis, fibrocystic kidney disease, hearing loss, anosmia, obesity, polydactyly and skeletal dysplasia. The precise clinical diagnosis, which depends on the constellation of phenotypes occurring in a given patient (Badano et al., 2006; Hildebrandt et al., 2011; Waters and Beales, 2011), can be challenging to establish given the important genetic and phenotypic overlap between individual disorders and the prominent phenotypic variability between individuals.

Ciliopathies are genetically heterogeneous disorders, typically inherited in a recessive manner, where bi-allelic mutations in one of many different genes can cause each disorder (Tobin and Beales, 2009). On the other hand, many ciliary genes can cause more than one ciliopathy disorder when mutated. Given this high genetic heterogeneity and the prominent phenotypic variability, it remains very challenging to predict the clinical outcome for a given individual and only very few genotype-phenotype correlations have been established in patients with ciliopathies (Mougou-Zerelli et al., 2009; Bachmann-Gagescu et al., 2015a), underscoring our still limited understanding of the mechanisms leading from mutations in ciliary genes to human disease phenotypes.

**FIGURE 1:**
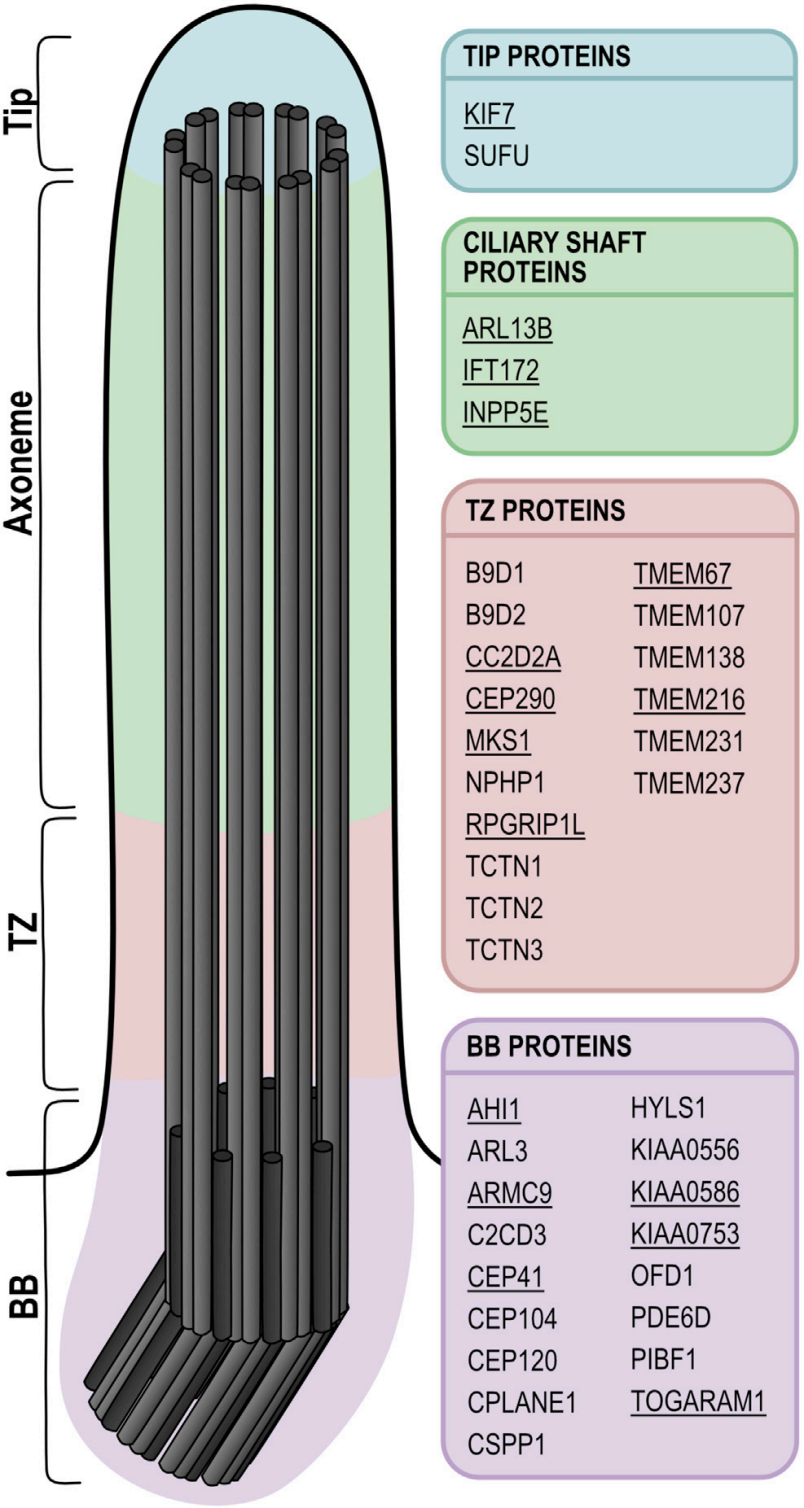
Schematic of a primary cilium showing the localization of JBTS proteins. Underlined proteins have at least one zebrafish mutant model available. While in this schematic only the main localization for each protein is indicated, it has been shown that many of these proteins can localize to several distinct ciliary subcompartments, which could be explained by dynamic localization and/or tissue-specific functions. In the ciliary shaft, IFT172 is part of the IFT-B complex, whereas ARL13B and INPP5E associate with the ciliary membrane. The subciliary localization of FAM149B1 is not known. BB: basal body; TZ: transition zone.

Joubert syndrome (JBTS) is a prototypical ciliopathy defined by a pathognomonic cerebellar and brainstem malformation called the “Molar Tooth Sign” (MTS), which is visible on axial brain magnetic resonance imaging (MRI). Clinically, the disorder is characterized by cerebellar ataxia, hypotonia, abnormal eye movements, and respiratory rhythm regulation disturbance (Romani et al., 2013). About 60% of JBTS patients display additional extra-CNS phenotypes such as retinal degeneration, kidney cysts, coloboma, polydactyly, liver fibrosis or encephalocele with high variability (Bachmann-Gagescu et al., 2015a). To date, 39 JBTS-associated genes, whose protein products localize to various compartments of the primary cilium, have been identified and are summarized in Table 1 and Figure 1 (Parisi, 2019; Bachmann-Gagescu et al., 2020). Mutations in several of these genes can also cause the more severe Meckel syndrome, which is characterized by encephalocele, polydactyly, cystic kidneys, hepatic fibrosis and other malformations, generally leading to fetal lethality (Hartill et al., 2017). In fact, based on the high degree of allelism, the two disorders are thought to represent extremes of the same disease spectrum.

**TABLE 1:** Phenotypes of JBTS zebrafish morphant (MO) and mutant models.

JBTS gene	Zebrafish orthologue	Conservation similarity/identity	Model(s)	Larval body curvature	Laterality defects	Hydrocephalus	Pronephric cysts	Otolith defects	Smaller eyes	Retinal dystrophy	CE defects	RNA rescue	Adult scoliosis	References
*AHI1*	*ahi1* ^*§*^	67 /50	MO	+	+	+	+	+	+	+	+	+	NA	MO: Simms et al. (2012), Elsayed et al. (2015), Zhu et al. (2019) / mut: Lessieur et al. (2017), Zhu et al. (2019)
			TALEN *iri46*, CRISPR	+	NA	-	+	-	+/-	+	-	+	+	
*ARL13B*	*arl13b* ^*§*^	75 / 60	MO	+	+	NA	+	NA	NA	NA	+	+	NA	MO: Sun et al. (2004), Duldulao et al. (2009), Zhu et al. (2020) / mut: Golling et al (2002), Cantagrel et al. (2008), Duldulao et al. (2009), Song et al. (2016), Zhu et al. (2020)
			ENU *hi459*	+	-	NA	+	NA	NA	+	NA	+	NA	
ARL3	*arl3a*	97 / 95
	*arl3b*	98 / 95
*ARMC9*	*armc9* ^*§*^	72 / 58	CRISPR *zh502, zh503, zh504, zh505*	-	-	NA	+	NA	NA	NA	NA	NA	+	Latour et al. (2020)
*B9D1*	*b9d1*	94 / 83	MO	NA	NA	NA	NA	NA	NA	NA	NA	NA	NA	Zhao and Malicki (2011)
*B9D2*	*b9d2*	86 / 76	MO	+	NA	NA	-	NA	NA	+	NA	+	NA	Dowdle et al. (2011), Zhao and Malicki (2011)
*C2CD3*	*c2cd3*	58 / 43
*CC2D2A*	*cc2d2a* ^*§*^	74 / 58	ENU *w38* *	+	NA	NA	+	NA	-	+	NA	NA	+	Gorden et al. (2008), Owens et al. (2008), Bachmann-Gagescu et al. (2011), Stawicki et al. (2016)
*CEP41*	*cep41*	72 / 59	MO	+	+	+	NA	+	+	NA	NA	+	NA	MO: J. E. Lee et al. (2012), Patowary et al. (2019), Ki et al. (2020) / mut: Ki et al. (2020)
			CRISPR *skk1*	NA	NA	NA	NA	NA	NA	NA	NA	NA	NA	
*CEP104*	*cep104*	68 / 52	MO	+	+	NA	NA	NA	NA	NA	NA	+	NA	MO and mut: Frikstad et al. (2019)
			CRISPR F0	+	+	NA	NA	NA	NA	NA	NA	NA	NA	
*CEP120*	*cep120*	74 / 59	MO	+	NA	+	+	+	+	NA	NA	+	NA	Shaheen et al. (2015)
*CEP290*	*cep290* ^*§*^	77 / 58	MO	+	+	+	+	+	+	+	NA	+	NA	MO: Sayer et al. (2006), Schäfer et al. (2008), Baye et al. (2011), Murga-Zamalloa et al. (2011), Cardenas-Rodriguez et al. (2021) / mut: Stawicki et al. (2016), Lessieur et al. (2019), Cardenas-Rodriguez et al. (2021)
			Tilling *fh297*, TALEN *fh378*, CRISPR fb208*	+	-	NA	+/-	-	NA	+	NA	NA	+	
*CPLANE1*	*-*	-
*CSPP1*	*cspp1a*	57 / 40	MO	+	NA	+	+	NA	NA	-	NA	NA	NA	Tuz et al. (2014)
	*cspp1b*	49 / 34	MO	+	NA	+	+	NA	NA	-	NA	NA	NA	Tuz et al. (2014)
*FAM149B1*	*fam149b1*	56 / 39
*HYLS1*	*-*	-
*IFT172*	*Ift172* ^*§*^	88 / 75	MO	+	NA	+	+	+	NA	+	NA	+	NA	MO: Sun et al. (2004), Lunt et al. (2009), Halbritter et al. (2013), Bujakowska et al. (2015), Bergboer et al. (2018), Eisa-Beygi et al. (2018) / mut: Amsterdam et al. (1999), Sun et al. (2004), Gross et al. (2005), Lunt et al. (2009), Sukumaran and Perkins (2009), Eisa-Beygi et al. (2018)
			Retroviral insertion *hi2211*	+	NA	NA	+	NA	NA	+	NA	NA	NA	
*INPP5E*	*inpp5e* ^*§*^	70 / 56	MO	+	NA	+	+	NA	+	+	NA	+	NA	MO: Luo et al. (2012), Xu et al. (2017)
			CRISPR	+	NA	NA	+	NA	NA	NA	NA	NA	NA	mut: Xu et al. (2017)
*KIAA0556/KATNIP*	*katnip*	80 / 69	MO	+	NA	NA	NA	NA	NA	NA	NA	+	NA	Roosing et al. (2016)
*KIAA0586/TALPID3*	*talpid3* ^*§*^	50 / 33	ZFN i262, i263, i264*	+	+	NA	+	NA	NA	+	NA	NA	NA	Ben et al. (2011), Ojeda Naharros et al. (2018)
*KIAA0753/OFIP*	*ofip*	49 / 34	ENU *sa22657*	+	NA	NA	NA	NA	NA	NA	NA	NA	NA	Hammarsjö et al. (2017)
*KIF7*	*kif7* ^*§*^	69 / 57	MO	NA	+	NA	NA	NA	NA	NA	NA	+	NA	MO: Tay et al. (2005), Wilson et al. (2009), Putoux et al. (2011) / mut: Maurya et al. (2013), Lewis et al. (2017), Terhune et al. (2021)
			ZFN i271, i272*, CRISPR *mw406*, CRISPR *co63*	+	+/-	-	NA	NA	NA	+	NA	+	+/-	
*MKS1*	*mks1*	40 / 26	MO	NA	NA	NA	NA	NA	NA	NA	+	+	NA	MO: Leitch et al. (2008)
			CRISPR *w152*	NA	NA	NA	NA	NA	NA	NA	NA	NA	NA	mut: Stawicki et al. (2016)
*NPHP1*	*nphp1*	66 / 46	MO	+	-	NA	+	NA	NA	NA	+	+	NA	Slanchev et al. (2011), Lindstrand et al. (2014)
*OFD1*	*ofd1*	60 / 40	MO	+	+	+	NA	+	NA	NA	+	NA	NA	Ferrante et al. (2009), Lopes et al. (2011)
*PDE6D*	*pde6b*	98 / 91	MO	NA	NA	NA	NA	NA	+	-	NA	+	NA	Thomas et al. (2014)
*PIBF1*	*pibf1*	82 / 66
*RPGRIP1L*	*rpgrip1l* ^*§*^	72 / 54	MO	+	+	+	NA	NA	NA	NA	+	+	NA	MO: Khanna et al. (2009), Mahuzier et al. (2012) / mut: Vesque et al. (2019)
			CRISPR F2	-	-	-	-	NA	NA	-	NA	NA	+	
*SUFU*	*sufu*	90 / 83	MO	NA	NA	NA	NA	+	+	NA	NA	NA	NA	Koudijs et al. (2005), Maurya et al. (2013)
*TCTN1*	*tctn1*	57 / 39	-											
*TCTN2*	*tctn2*	53 / 38	MO	NA	+	NA	NA	NA	NA	NA	NA	NA	NA	Liu et al. (2018)
*TCTN3*	*-*	-
*TMEM67*	*tmem67* ^*§*^	75 / 59	MO	+	NA	+	+	+	+	NA	+	+	NA	MO: Adams et al. (2012), Leightner et al. (2013), Lee et al. (2017), Stayner et al. (2017) / mut: Zhu et al. (2021)
			TALEN *e3*	+	NA	-	+	NA	NA	NA	-	NA	+	
*TMEM107*	*tmem107*	76 / 61
*TMEM138*	*tmem138*	80 / 68	MO	+	+	-	NA	NA	NA	NA	+	NA	NA	Lee J. H. et al. (2012)
*TMEM216*	*tmem216* ^*§*^	77 / 58	MO	+	+	+	NA	NA	NA	NA	+	+	NA	MO: Valente et al. (2010), Lee J. H. et al. (2012) / mut: Liu et al. (2020)
			CRISPR *sny∆175, snyR8∆60*	NA	NA	-	-	NA	NA	+	NA	NA	NA	
*TMEM231*	*tmem231*	78 / 58
*TMEM237*	*tmem237a*	68 / 53	MO	NA	NA	NA	NA	NA	NA	NA	+	+	NA	Huang L. et al. (2011)
	*tmem237b*	68 / 52	MO	NA	NA	NA	NA	NA	NA	NA	+	+	NA	Huang L. et al. (2011)
*TOGARAM1*	*togaram1* ^*§*^	54 / 38	CRISPR *zh508, zh510*	+	-	NA	+	NA	NA	NA	NA	NA	+	Latour et al. (2020)

List of bona fide JBTS genes ordered alphabetically (genes with currently limited evidence are not included). Conservation between the zebrafish and the human gene is shown at the amino acid level (similarity / identity). To determine conservation, available sequence information from genome assemblies GRCz11 (zebrafish) and GRCh38.p13 (human) were used, apart for inpp5e, armc9 and togaram1, for which more complete sequence generated in our laboratory was available. §: Genes for which the zebrafish mutant model was described with sufficient information to allow inclusion for the comparisons shown in Figure 5. MO models are always listed first, mutant models second for genes where both are published. The references for each gene are separated according to model type (MO: morphant, mut: mutant).

CE: conversion-extension. The absence or presence of a defect in ciliary morphology in the different organs is shown in Supplementary Table S1. **+**: phenotype present, **-**: phenotype absent, NA: not available/not described. *maternal-zygotic mutant available.

Many JBTS proteins collaborate in multiprotein complexes at the ciliary transition zone (Garcia-Gonzalo et al., 2011; Sang et al., 2011; Williams et al., 2011), and JBTS has been proposed to be caused specifically by transition zone dysfunction (Shi et al., 2017). However many of the more recently identified JBTS-associated genes encode proteins that don’t localize to the transition zone but to other ciliary subcompartments, with normal transition zone integrity (Kobayashi et al., 2014; Schwarz et al., 2017; Van De Weghe et al., 2017; Latour et al., 2020) (Figure 1). Thus, it remains elusive what distinguishes JBTS from other ciliopathies. Moreover, despite progress in understanding the function of primary cilia, the pathomechanism underlying ciliopathies remains partly unsolved. Given the well-established link between primary cilia and Hh signaling, aberrant regulation of this pathway is a likely mechanism explaining certain phenotypes such as polydactyly, skeletal abnormalities or craniofacial phenotypes. In contrast, the retinal phenotypes are unlikely to be explained by aberrant Hh signaling regulation and the cause for the CNS, kidney or liver phenotypes remains controversial, with multiple lines of evidence pointing towards a variety of pathomechanisms. Generating robust and well-characterized models for ciliopathies and JBTS therefore remains crucial, in order to elucidate the link leading from mutations in ciliary genes to the various ciliopathy-associated phenotypes.

## Zebrafish as a Model Organism for Ciliopathies and Joubert Syndrome

In the past two decades, the zebrafish (*Danio rerio*) has become a popular model to study human disease and gene function due to its practical advantages, including large numbers of externally developing transparent embryos. Rapid development leads to free swimming and behaving larvae with fully functioning organ systems within 5 days post fertilization (dpf). Transparency of embryos allows for unique imaging conditions, including in live animals, and the available genetic toolbox allows for efficient generation of transgenic and mutant lines (Kawakami, 2007; Santoriello and Zon, 2012; Howe et al., 2013). The zebrafish genome has a size of ∼1,412 gigabases (Gb) distributed among 25 chromosomes with 26,206 protein-coding genes (Howe et al., 2013). About 71% of the ∼20,500 human protein-coding genes have orthologues in the zebrafish genome, with 82% of human disease genes having a zebrafish orthologue (Howe et al., 2013). The higher number of genes in zebrafish compared to humans is explained by the teleost-specific whole genome duplication event that occurred about 320–350 million years ago (Glasauer and Neuhauss, 2014). While some of the resulting zebrafish paralogues were maintained, many duplicated genes were lost shortly after the duplication event (Sassen and Köster, 2015). Interestingly, genes that are involved in neuronal function often retained both paralogues (Glasauer and Neuhauss, 2014). In contrast, ciliary genes tend to not have more than one zebrafish orthologue. Specifically, of 39 JBTS-associated genes, only three (*TMEM237, CSPP1* and *ARL3*) have two paralogues in zebrafish (Table 1). All JBTS-genes apart from *CPLANE1*, *TCTN3*, *HYLS1* and *TOGARAM1* have an annotated zebrafish orthologue in the current genome assembly (GRCz11). However, this genome annotation remains incomplete to date and it is possible that zebrafish orthologues for these genes do exist. In fact, the zebrafish *togaram1* gene for instance was present in earlier annotations and has vanished in the most recent genome annotation. However, we have cloned this gene from zebrafish larvae and shown by synteny analysis that it is the true orthologue of human *TOGARAM1.* On average, JBTS zebrafish orthologues show a similarity of 71% and an identity of 57% (Table 1) with the human proteins, making the zebrafish an excellent model system to study Joubert syndrome (Howe et al., 2013).

**FIGURE 2:**
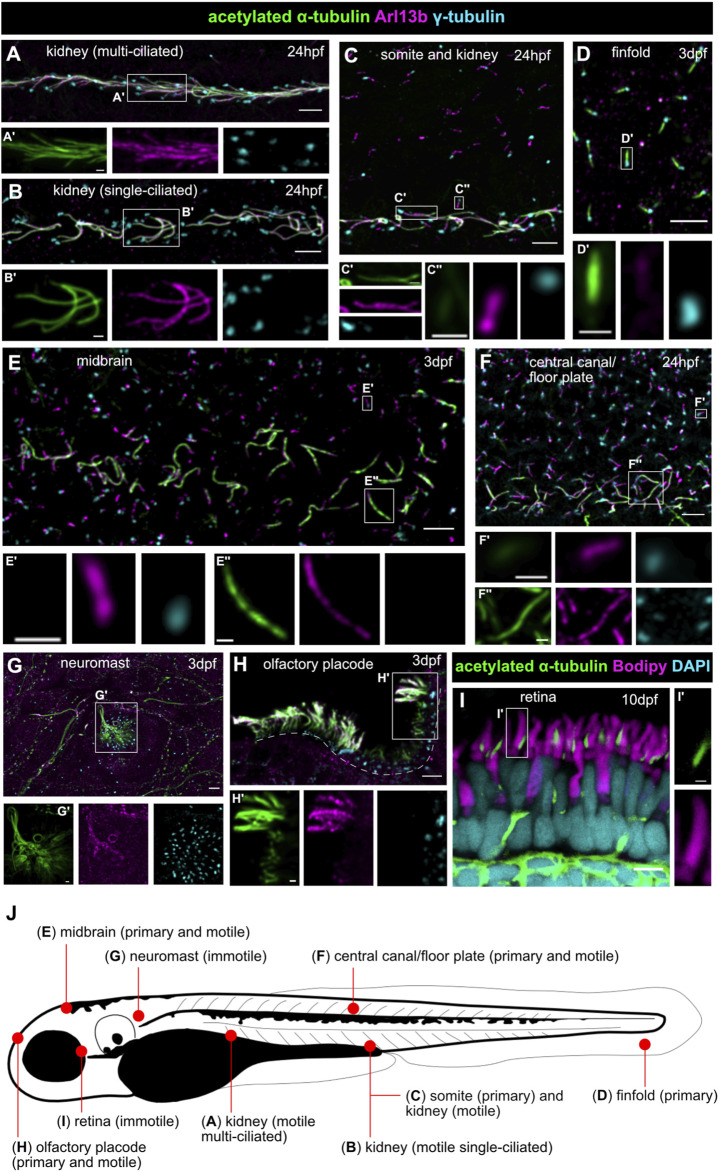
Various types of motile and immotile cilia in zebrafish show distinct acetylated tubulin or Arl13b signal patterns. **(A,B)** Cilia in the pronephros are all motile, but multiple **(A)** or single **(B)** on tubular cells in the different portions of the nephron. **(C)** Long strongly acetylated α-tubulin positive motile kidney cilia **(C’)** and shorter primary somite cilia **(C’’)** with weaker acetylated α-tubulin signal. **(D)** Primary cilia on the finfold with very weak Arl13b signal. **(E)** Short primary neuronal cilia without visible acetylated α-tubulin signal (at least with these imaging settings) **(E’)** and longer motile midbrain cilia strongly positive for acetylated α-tubulin at the same imaging conditions **(E’’). (F)** Short primary cilia in the central canal **(F’)** with weak acetylated α-tubulin signal and longer acetylated α-tubulin positive motile floor plate cilia **(F’’)**. **(G)** Immotile neuromast sensory cilia**. (H)** Long motile cilia at the border of the olfactory placode and shorter Arl13b-poor primary cilia in the center. Dotted lines mark the olfactory placode for orientation. **(I)** The outer segment of retinal photoreceptors (marked in magenta with bodipy) is a highly modified primary cilium; note the presence of the axoneme on the side of the outer segment, marked in green by acetylated α-tubulin. **(J)** Schematic overview of a zebrafish larva showing localization of the various cilia types. All images [except **(I)**] are whole mount immunofluorescence using anti-acetylated α-tubulin (green), anti-Arl13b (magenta) and anti-γ-tubulin (cyan), imaged using a spinning disk microscope at the following stages: 24 hpf **(A–C,F)** and 3 dpf **(D–H)**. Lateral views in **(A–D,F,G)**; dorsal view in **(E,H)**. **(I)** Immunofluorescence on a retinal cryosection at 10 dpf with anti-acetylated α-tubulin (green), bodipy (magenta) and DAPI (cyan) imaged by confocal microscopy. Scale bars are 5 μm in the overview pictures and 1 μm in the insets.

One strength of the zebrafish as a model for human ciliopathies (including JBTS) is the variety of specialized cilia types present in this animal, similar to the human situation. Indeed, various types of cilia are found on virtually all zebrafish cells, including among others cells lining the Kupffer’s vesicle (equivalent of the embryonic node), neuroephithelial cells in the developing brain and those lining the brain ventricles and the central canal, cells of the olfactory placode, in the otic placode and later in the ear, neuromasts from the lateral line organ (sensing fluid flow), epithelial cells in kidney tubules or photoreceptors in the retina (Figure 2). In both human and zebrafish retinal photoreceptors, primary cilia have become highly specialized, forming what are called “outer segments” to perceive incoming light signals (Kennedy and Malicki, 2009; Bachmann-Gagescu and Neuhauss, 2019). These outer segments are composed of multiple stacks of membrane disks or folds, arranged around a typical microtubule-based axoneme. The transition zone is called connecting cilium in photoreceptors and links the large outer segment ciliary compartment to the inner segment, which is part of the cell body of the photoreceptor. This arrangement is perfectly conserved between zebrafish and humans. However, the distinction between primary immotile and motile cilia is somewhat blurred in the zebrafish compared to human in other organ systems, since some cilia which are immotile in humans (such as those present on renal tubular cells) are single but motile in the corresponding cell type in zebrafish larvae (Kramer-Zucker et al., 2005). The olfactory placode of zebrafish larvae contains both motile cilia on the periphery and immotile cilia in the center (Reiten et al., 2017). Likewise, the Kupffer’s vesicle contains a mix of motile and immotile cilia. The cilia most frequently analyzed in currently existing ciliopathy models are those present on cells lining the brain ventricles, the olfactory placode, the kidney tubules, the central canal, the ear and the Kupffer’s vesicle, which are single but mostly motile cilia. In fact, variation between cilia in different tissues has been little analyzed so far in the zebrafish. Typically used ciliary markers for which reliable antibodies exist in this model system are anti-Arl13b [zebrafish-specific antibody generated by Z. Sun (Duldulao et al., 2009)] and anti-acetylated tubulin (commercially available). When staining a range of primary and motile cilia in various zebrafish larval tissues, important variations of the relative intensities of these two signals are observed (Figure 2). A comprehensive analysis of tissue-specific differences in Arl13b, acetylated tubulin, polyglutamylated tubulin and other available ciliary markers remains to be performed.

**FIGURE 3:**
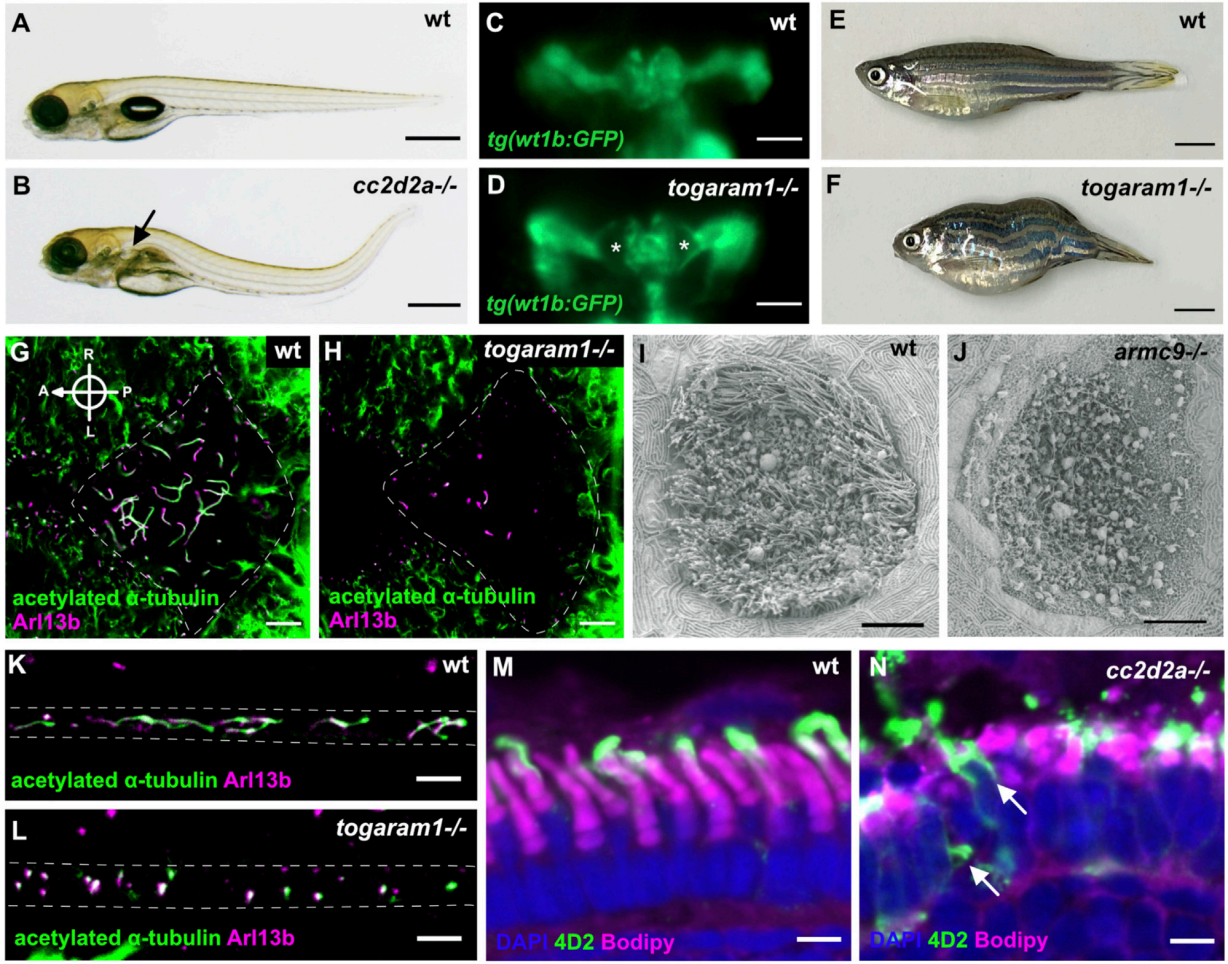
Examples of typical ciliopathy phenotypes in various zebrafish JBTS mutants. **(A,B)** 5 dpf old wildtype larva with straight body **(A)**. Curved body shape and kidney cysts (black arrow) in *cc2d2a−/−* **(B)**. **(C,D)** Transgenic *tg(wt1b:GFP)* line highlighting the pronephros of 3 dpf wildtype **(C)** and *togaram1−/−* **(D)** larvae showing an enlargement of the proximal tubules close to the glomerular region (=kidney cysts) marked by asterisks in the mutant. Dorsal view, rostral to the top. **(E,F)** Adult wildtype zebrafish **(E)** with a straight body axis compared to scoliosis in *togaram1−/−* **(F)**. **(G,H)** Whole mount immunohistochemistry of forebrain ventricular cilia in 3 dpf old wildtype **(G)** and *togaram1−/−* **(H)** larvae showing shorter and fewer cilia in mutants with decreased acetylation (green, acetylated α-tubulin). Dotted lines mark the border of the ventricular space for orientation. Note that green signal outside of the ventricle stems from axons which are rich in acetylated α-tubulin. **(I,J)** Scanning electron microscopy (SEM) image of the olfactory placode cilia in 5 dpf old wildtype **(I)** and *armc9−/−* **(J)** larvae showing almost absent cilia in the olfactory placode of the mutant. **(K,L)** Immunohistochemistry of kidney cilia in 3 dpf old wildtype **(K)** and *togaram1−/−* **(L)** larvae showing shorter cilia in mutants. Dotted lines mark border of the pronephric tubule for orientation (lateral view with rostral to the left). **(M,N)** Immunohistochemistry on cryosections of 5 dpf wt **(M)** and *cc2d2a−/−* **(N)** larvae showing normal retinal lamination but shortened and dysmorphic outer segments (marked by bodipy in magenta) and mislocalization of opsins to the photoreceptor cell body (4D2 antibody in green, arrows) in mutants. Scale bars are 500 μm **(A,B)**, 50 μm **(C,D)**, 5 mm **(E,F)**, 10 μm **(G–J)** and 5 μm **(K–N)**.

**FIGURE 4:**
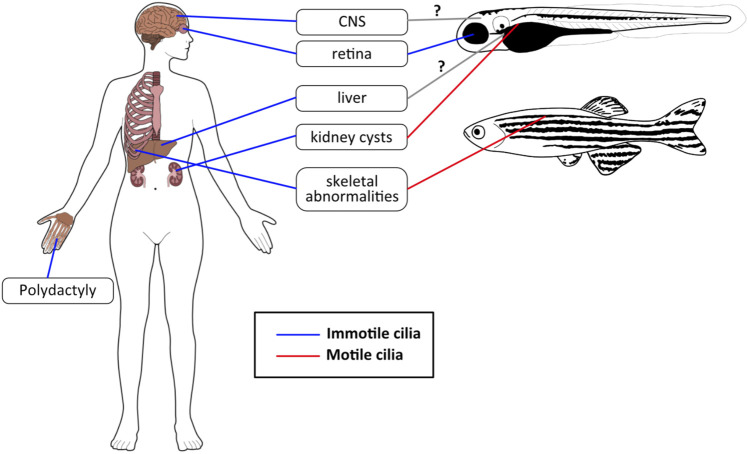
JBTS phenotypes in humans and zebrafish. The most common JBTS phenotypes seen in humans have analogous phenotypes in zebrafish, although these are sometimes caused by motile cilia dysfunction rather than defects in primary cilia. Blue lines: immotile/primary cilia, red lines: motile cilia, gray lines with question mark (?): not clear yet whether zebrafish JBTS models display a phenotype in these organs (and which types of cilia would be involved).

Beyond the high conservation of ciliary genes between zebrafish and human and the variety of cilia types, the strong morphological conservation between tissues and/or functional conservation of various organs validate the use of this model system to study human ciliopathies. This conservation is particularly well illustrated in the retina, which has very similar tissular morphology and displays similar cell types, including rod and cone photoreceptors (Bibliowicz et al., 2011; Angueyra and Kindt, 2018), as in humans. The larval zebrafish pronephros represents a simplified version of the human nephron, the functional unit of a human kidney: the larval pronephros is formed by a single fused glomerulus with two symmetrical tubule systems converging in the cloaca and displays the same cell types in its glomerulus and the same specific segments in its tubular system as in human nephrons (Wingert and Davidson, 2008; Morales and Wingert, 2017). Being a vertebrate, the zebrafish is also an excellent model for scoliosis and other spinal anomalies (Boswell and Ciruna, 2017). Consequently, zebrafish harboring mutations in ciliopathy genes display typical ciliopathy phenotypes such as cystic kidneys or retinal dystrophy and degeneration, often accompanied by spinal curvature (Figures 3, 4). A further advantage of the zebrafish system is the possibility to perform functional assays at the organismal level to quantify the function of various organs. For example, electroretinograms or the oculo-kinetic response (OKR) are well-established assays to evaluate the visual function of zebrafish (Fleisch and Neuhauss, 2006).

The genetic toolbox available in zebrafish to model human disorders, in this case ciliopathies, includes efficient mutagenesis and transgenesis (Kawakami, 2007; Sassen and Köster, 2015). Currently available zebrafish models for ciliopathies (including JBTS) were identified either in forward genetic screens (e.g., *cc2d2a* ^*w38*^ and *arl13b* ^*hi459*^) or, more recently, were generated using reverse genetics approaches. The latter include TILLING (e.g., *cep290* ^*fh297*^), where a DNA library from ENU-mutagenized male zebrafish is screened for mutations in genes of interest (Moens et al., 2008), or directed mutagenesis of the gene of interest using Zinc-finger (*kif7* ^*i271/i272*^ and *talpid3* ^*i262/i263/i264*^), TALEN (*ahi1* ^*iri46*^, *cep290* ^*fh378*^ and *tmem67* ^*e3*^) or most recently CRISPR-Cas mediated nucleases (e.g., *armc9* ^*zh505*^, *togaram1* ^*zh510*^ and *kif7* ^*mw406*^) (Urnov et al., 2010; Huang P. et al., 2011; Zhang et al., 2014). The efficiency of CRISPR mutagenesis is such that phenotypes are often already observed in the F0 injected fish, which are mosaic for various induced mutations (and unmutated cells) (Shah et al., 2015). In parallel to knocking out genes of interest at the genomic level, a commonly used technique in the zebrafish is the transient oligonucleotide-based knockdown using morpholinos (MOs). The advantage of MOs lies in their rapid application as the phenotypes can be studied directly in the injected embryo (Nasevicius and Ekker, 2000). This represents an important advantage over generating stable mutants given the long (3-month) generation time for zebrafish. A further advantage of using MOs is that they avoid the potential problem of phenotypic rescue through maternally deposited mRNA in the egg (Abrams and Mullins, 2009). This can be circumvented in mutants by generating maternal zygotic mutants (offspring of a homozygous mutant mother) which is however a time-consuming endeavor. In addition, the level of gene knockdown can be titrated using morpholinos, which can represent another advantage in case complete loss-of-function is lethal, for example. On the other hand, the limitation of using MOs lies in the frequent off-target effects, which need to be carefully controlled for, and in the limited duration of the knockdown effect, making it impossible to study adult phenotypes (Stainier et al., 2017). Transgenesis approaches, in which a fluorophore-tag is added to a protein of interest such as the ciliary protein Arl13b or the centrosomal protein Centrin, allow visualization of cilia or centrosomes (Borovina et al., 2010; Malicki et al., 2011).

The ubiquitous presence of specialized cilia on a variety of zebrafish cells, together with the strong conservation of organs and genes between human and zebrafish, and the technical toolbox available make the zebrafish a powerful model to study ciliopathies such as JBTS. Here, we review the various zebrafish models for JBTS published to date, comparing morphants with mutants for the same genes, as well as different mutants in various JBTS genes with each other, in an attempt to identify commonalities and discrepancies. By summarizing the insights gained from zebrafish models, we hope to reach a deeper understanding of the function of JBTS genes in ciliary biology and of the pathomechanism underlying this disorder.

## Comparison Between Zebrafish Models for Joubert Syndrome

Currently, 39 genes have been identified to cause JBTS when mutated (Table 1), of which 29 (74%) have at least one zebrafish model available (morphant or mutant model). For 16 genes (41%), a stable mutant model has been published (Figure 1), with several alleles generated for eight of these: *ahi1, armc9, cc2d2a, cep290, kif7, kiaa0586/talpid3, tmem216* and *togaram1* (Table 1). Since identification of JBTS-causing genes and subsequent generation of zebrafish models occurred in parallel to the evolution of genome editing tools, this explains the broad variety of methods used to generate zebrafish models: ENU (*n* = 3), TILLING (*n* = 1), retroviral insertion (*n* = 1), ZFN (*n* = 2), TALEN (*n* = 3), CRISPR (*n* = 11), MO (*n* = 24) (Table 1). MOs have been used most commonly to generate JBTS zebrafish models given the advantages explained above (ease of use and short time to results). On the other hand, the CRISPR/Cas9 system has been used most frequently for permanent genome editing, despite the fact that it is the most recent technique, underscoring its efficiency to generate stable zebrafish mutant lines.

### Phenotypes Observed in Zebrafish Morphant and/or Mutant Models of Joubert Syndrome

**FIGURE 5:**
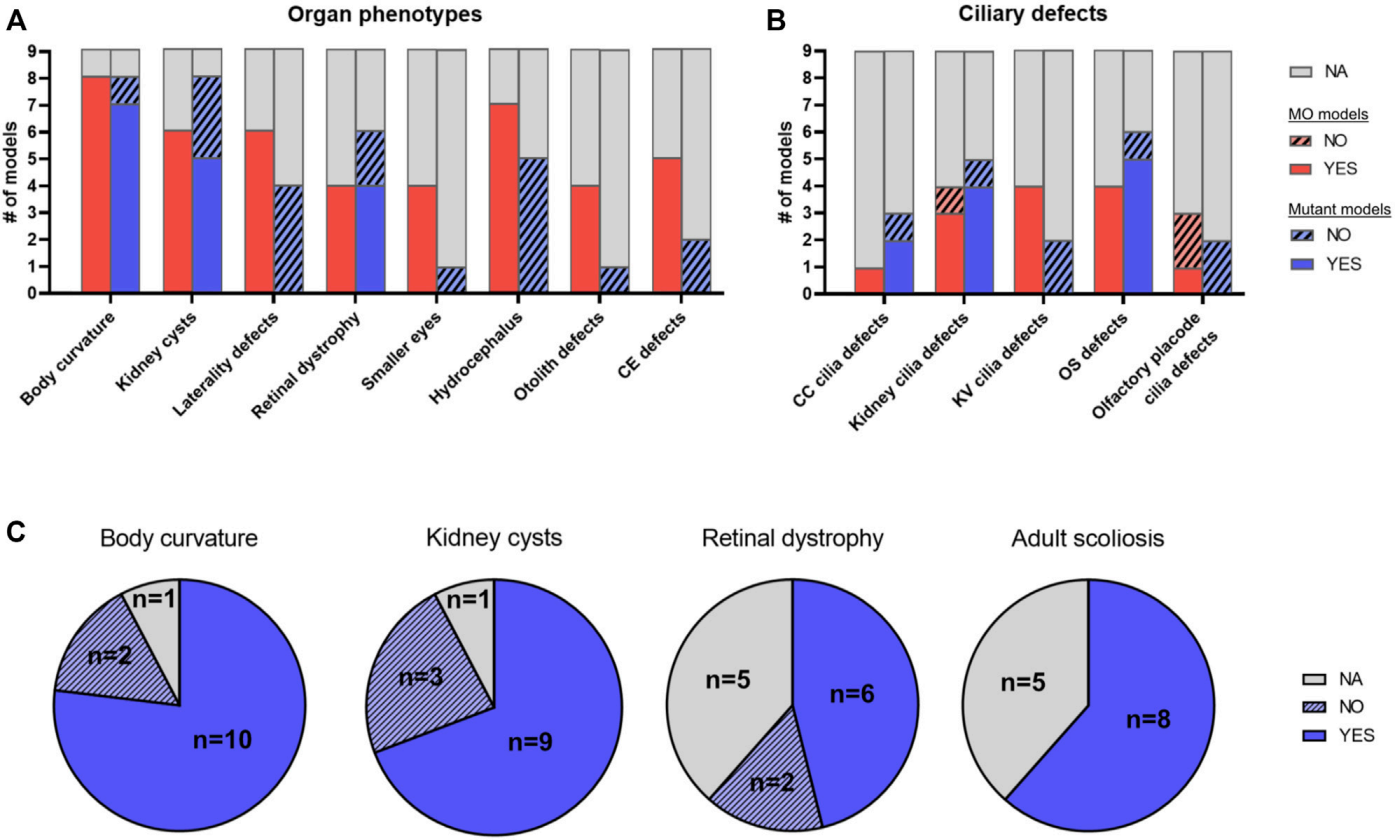
Phenotypic comparison between JBTS zebrafish models. **(A,B)** Comparison of morphant and mutant models for nine different JBTS genes (*ahi1, arl13b, cep290, ift172, inpp5e, kif7, rpgrip1l, tmem67* and *tmem216*) where sufficient phenotypic information was available: Histogram plot illustrating the number of models displaying a given organ phenotype **(A)** or a given ciliary defect **(B).** Red: MO models, blue: mutant models. Note how some phenotypes such as laterality defects, hydrocephalus or convergence-extension defects are often described in morphant models but not in mutant models. **(C)** Comparison of mutant zebrafish models for 13 JBTS genes with sufficient phenotypic information [as indicated in Table 1; the 13 mutant lines analyzed here include the nine lines analyzed in **(A,B)** plus *armc9, cc2d2a, talpid3* and *togaram1*, for which no morphant phenotype has been described]. Pie chart plots showing the proportion of mutant models displaying each of the four most common ciliopathy phenotypes seen in zebrafish (body curvature, kidney cysts, retinal dystrophy and adult scoliosis). YES: model has the phenotype, NO: model does not have the phenotype, NA: not available/not described. Note that some mutants are included in both sets of comparisons in **(A)** and **(B)** (Table 1). CC: central canal; CE: conversion-extension; KV: Kupffer’s vesicle; MO: morphant; OS: outer segment.

We first sought to compare MO models to the corresponding mutant models for the same genes, in order to investigate consistency and potential discrepancies of occurring phenotypes (Robu et al., 2007; Eisen and Smith, 2008). We found nine JBTS-causing genes that have both a MO and a mutant model with sufficient phenotypic characterization (Table 1; Supplementary Table S1). In general, MO models tend to exhibit more phenotypes than their corresponding mutant models (Figure 5). In particular, phenotypes such as laterality defects, hydrocephalus, otolith defects, small eyes, conversion-extension (CE) defects or Kupffer’s vesicle (KV) cilia defects were described only in MO zebrafish models but not in mutant JBTS models (Figures 5A,B). Such phenotypes may represent off-target toxicity effects and may not be specific to downregulation of the targeted gene. Alternatively, the additional phenotypes seen only in morphants could be explained by compensation mechanisms occurring only in mutants (Rossi et al., 2015; El-Brolosy et al., 2019; Cardenas-Rodriguez et al., 2021) and/or by rescue of the phenotype in zygotic mutants through presence of maternally deposited mRNA and/or protein in the egg. However, for a small number of mutants, maternal zygotic mutants were generated and these still did not present with CE defects, laterality defects, smaller eyes or hydrocephalus (e.g., *cc2d2a*, *kif7*, *talpid3* and *cep290*). Unfortunately, control experiments are lacking for a number of morphants, such that non-specific off-target toxicity defects cannot be ruled out. On the other hand, some phenotypes including retinal defects, kidney cysts or body curvature are present in both MO and mutant models, supporting specificity of these phenotypes caused by ciliary dysfunction (Figure 5). However, a lack of systematic description of all possible phenotypic features in most models somewhat limits the comparison between MO and mutant models.

### Comparison Between Zebrafish Mutants in Joubert Syndrome Genes

Focusing only on stable mutant lines described, we next compared the phenotypes present in the various JBTS mutants with each other. Since all these genes cause the same human disorder, we were expecting to find similar zebrafish phenotypes between the different mutants. Unfortunately, in most cases, a systematic analysis of all possible cilia-related phenotypes is lacking for the described JBTS zebrafish models (Table 1; Supplementary Table S1), which somewhat limits this analysis. Indeed, often only a specific phenotype of interest was investigated and described. Phenotypic characterization was mostly conducted on zygotic mutant zebrafish since adult zebrafish harboring mutations in JBTS-genes often exhibit reduced viability, scoliosis and reduced fertility (Mytlis et al., 2022), making the generation of maternal zygotic mutants *via* natural matings challenging (requires *in vitro* fertilization and/or generation of germline mosaic fish) (Ben et al., 2011).

We identified a total of 13 mutant zebrafish models with sufficient phenotypic characterization to be included in the following analysis (Table 1; Figure 5; Supplementary Table S1). For several genes, more than one stable mutant line has been generated using various genome editing tools. While in most cases the different alleles for a JBTS given gene were indistinguishable from each other, distinct phenotypes were occasionally described. For example, for *cep290*, the *fh297* allele exceptionally displays pronephric cysts while these are not observed in the *fh378* and *fb208* alleles, and for *kif7*, *i271/i272* mutants do not display scoliosis as adults, while *co63* mutants do. Phenotypes seen most commonly in zebrafish JBTS mutants included larval body curvature, kidney cysts, retinal defects and scoliosis in adults (Figure 5). Notably, retinal and renal defects are also most common in individuals with JBTS in addition to the characteristic CNS malformation (Romani et al., 2013; Bachmann-Gagescu et al., 2015a). A cerebellar defect has been described in a single JBTS zebrafish model so far, namely for *arl13b* (Zhu et al., 2019). This work found that in *arl13b* morphant (and mutant) larvae, the number of cerebellar granule cells was reduced and the development of Purkinje cells was also affected, anomalies that were linked to defective WNT signaling. Of note, the described central nervous system phenotypes were relatively mild compared to the anomalies found in mouse models for *Arl13b* (Higginbotham et al., 2012; Suciu et al., 2021) or in human patients harboring *ARL13B* mutations (Cantagrel et al., 2008; Thomas et al., 2015). No other studies have described cerebellar anomalies in zebrafish JBTS models yet, whereby it remains unclear how thoroughly the central nervous system has been analyzed in the published zebrafish JBTS models to date. Morphological anomalies of cilia described most commonly in JBTS zebrafish models included shortened/lack of renal cilia and defective outer segments (OSs) (Supplementary Table S1).

## Discussion: Insights Into Ciliary Function and Joubert Syndrome Pathomechanisms Gained From Zebrafish Models

One puzzling question in JBTS disease mechanism is how dysfunction of so many genes, whose protein products localize to different subcompartments of the primary cilium, results in the same disorder characterized by a pathognomonic hallmark, the distinctive “Molar Tooth Sign” (MTS). Given the large number of other non-JBTS associated ciliopathy genes, another way of putting this question is to ask what sets JBTS-associated genes apart from other ciliary genes. On the other hand, despite the unifying brain malformation of JBTS, the prominent phenotypic variability among individuals with JBTS, in particular with respect to associated non-CNS phenoytpes, suggests distinct roles in ciliary biology, possibly explained by the specific subciliary localization of the proteins, and/or by tissue-specific functions for different JBTS-genes. By comparing the phenotypes of the different zebrafish mutants for JBTS we aimed at identifying commonalities and differences between JBTS genes.

Given the strong conservation of the retina between human and zebrafish and the almost constant retinal involvement in zebrafish ciliopathy mutants, most data are available on this organ system, with thoroughly studied mutants for *cc2d2a, talpid3, ift172* and *ahi1*. Interestingly, even though these four mutants all display some degree of retinal dystrophy, which in patients would be considered as “the same phenotype,” the underlying mechanism appears to be radically different. In the case of the *cc2d2a* ^*w38*^ mutant, the retinal dystrophy is caused by deficient fusion of incoming opsin-carrier vesicles at the ciliary base, leading to massive accumulation of vesiculo-tubular structures in the inner segment of photoreceptors and dysmorphic outer segments. The proposed model suggests that Cc2d2a, localizing at the connecting cilium (the equivalent of the transition zone in photoreceptors), plays a dual role in the last steps of Rab8-controlled opsin carrier vesicle trafficking (Bachmann-Gagescu et al., 2011; Ojeda Naharros et al., 2017): On the one hand, the vesicle fusion machinery at the periciliary membrane, including SNAREs SNAP25 and Syntaxin3, is disorganized in the absence of Cc2d2a; on the other hand, Cc2d2a provides a docking point for incoming vesicles through a chain of interactions involving Cc2d2a-Ninl-Mical3-Rab8 (Bachmann-Gagescu et al., 2015b). The centrosomal protein Ninl also binds to the cytoplasmic dynein motors that move vesicles along microtubules towards the ciliary base. In contrast to Cc2d2a, Talpid3 deficiency causes retinal dystrophy through a radically different mechanism. *talpid3* ^*i262-264*^ zebrafish mutants demonstrate retinal degeneration with photoreceptors entirely lacking outer segments, which is secondary to defects in basal body positioning and docking at the apical cell surface (Ojeda Naharros et al., 2018). The very first step of ciliogenesis, which involves Rab8-dependent fusion of the ciliary vesicle onto the mother centriole, appears to be disrupted by loss of Talpid3. Interestingly, the deficient outer segment formation in *talpid3* mutant zebrafish could be rescued by overexpression of a constitutively active form of Rab8. While the ciliogenesis and loss of basal body docking had been previously described in other model systems (Davey et al., 2006; Bangs et al., 2011), and the link to Rab8 had already been identified in cell culture (Kobayashi et al., 2014), the zebrafish experiments allowed to place Talpid3 upstream of Rab8 activation. As a third mechanism underlying photoreceptor dysfunction, studies of an *ift172* zebrafish mutant revealed a distinct function in outer segment formation. Mutant photoreceptors failed to complete extension of the cilium, after successful basal body docking and formation of the connecting cilium. Additionally, large accumulation of membranous material was found within the inner segment, suggesting that photoreceptors try to assemble outer segment material, but fail to extend the ciliary structure (Sukumaran and Perkins, 2009). Finally, the mechanism underlying the retinal dystrophy in *ahi1* mutants is not definitely determined, but the described dysmorphic outer segments, aberrant disk stacking/orientation and accumulation of vesicular material with normal connecting cilium morphology could indicate a similar role in vesicular fusion as for Cc2d2a (Lessieur et al., 2017). These studies of zebrafish retina revealed different molecular mechanisms leading to the same ciliopathy phenotype, namely retinal dystrophy. Whereas Talpid3 and Ift172 play a role in ciliogenesis, either in proper BB docking at the onset of ciliogenesis or later in outer segment extension, the role of Cc2d2a lies downstream of ciliogenesis, in organizing the last steps of opsin vesicle trafficking and fusion. In fact, the distinct ciliary defects observed in the different mutants are consistent with the localization of the respective proteins: Talpid3 localizes to the BB, which can explain defects in early ciliogenesis steps, Ift172 is part of the IFT machinery, required to transport the building blocks for the extension of the ciliary axoneme and Cc2d2a at the ciliary transition zone organizes the periciliary membrane and provides a docking point for incoming vesicles. Whether these specific roles for each of the proteins are conserved in other cell types than photoreceptors remains open for investigation. Indeed, photoreceptors display such highly specialized cilia, that a cell-type specific role for JBTS proteins is plausible here.

To investigate another aspect of ciliary function, the *talpid3* ^*i262-264*^ mutants were initially generated to evaluate the role of this protein in Hh signaling (Ben et al., 2011). Indeed, despite the solid body of evidence linking primary cilia to Hh signaling in mammalian cells, this link appeared less clear in zebrafish, probably in part because major CNS anomalies which are typically present in mouse mutants for ciliary genes, appear to be absent or too subtle to notice without in depth characterization in the corresponding zebrafish mutants, judging by the lack of such mentions in the literature (including for *talpid3* mutants). In contrast, the chick *talpid3* mutant was originally identified as a Hh-mutant, based on the characteristic phenotype including limb patterning defects and craniofacial anomalies (Davey et al., 2006). The *talpid3* ^*i262-264*^ zebrafish mutants lack cilia in all organs, consistent with the absent photoreceptor outer segments and with results from mouse and chick studies (Davey et al., 2006; Yin et al., 2009; Bangs et al., 2011). This absence of cilia was shown to result also in zebrafish in aberrant Hh signaling, as shown by expansion of the Hh-dependent muscle pioneer cells in the somites (Ben et al., 2011). Further confirming the involvement of cilia in zebrafish Hh signaling, the same group showed that zebrafish Kif7 directly interacts with Gli1 and Gli2a suggesting a function in sequestering Gli proteins in the cytoplasm (Maurya et al., 2013).

Interestingly, the *kif7* mutant generated in this study had no CNS patterning abnormality, which raises the possibility that this signaling pathway does not require cilia for CNS-patterning in zebrafish. Alternatively, functional redundancy between ciliary proteins may be more prominent in the zebrafish than in mammals, such that knock-out of more than one ciliary gene might be required to generate an obvious CNS phenotype, similar to what is observed in *C. elegans*, where double mutants are often required to observe strong ciliary anomalies (Williams et al., 2011). However, considering specifically the cerebellum, which is predominantly affected in individuals with JBTS, the importance of Hh signaling *per se* in zebrafish cerebellar development is still under debate (McFarland et al., 2008). Indeed, the Purkinje cells, which are an important source of SHH during mammalian and avian cerebellar development, appear not to secrete this morphogen in zebrafish. Since SHH is required for proliferation of cerebellar granule cells in mammals, this important difference may explain why no strong cerebellar phenotype has been published yet in zebrafish *kif7, talpid3* or other zebrafish JBTS-models. Nevertheless, a recent study did identify cerebellar eurydendroid cells as a source for SHH in the zebrafish cerebellum, such that a role for this pathway is still possible during cerebellar development in this organism (Biechl et al., 2016). Its link to primary cilia in development of the zebrafish cerebellum/CNS remains however an open question. An in-depth analysis of the CNS and specifically of the cerebellum in the available zebrafish mutants will be required to further tease apart the role of Hh signaling in cerebellar development in zebrafish and the role of primary cilia in regulating Hh signaling in the developing zebrafish cerebellum. To our knowledge, a single study has described some cerebellar anomalies in a zebrafish *arl13b* morphant (and mutant) model with a decrease in granule cells in the corpus cerebelli and alteration of expression levels of various cerebellar markers (Zhu et al., 2020). Interestingly, this phenotype was found to be linked to dysregulation of another key developmental signaling pathway, namely Wnt signaling, while Hh signaling was not investigated in this model. Further studies will be required to investigate the CNS phenotype of zebrafish JBTS mutants and to determine the role of primary cilia in developmental signaling in cerebellar/CNS development in this model system.

Such work would be of importance for understanding the pathomechanism underlying JBTS. Indeed, aberrant Hh signaling appears to be a possible common downstream consequence of dysfunction of many (if not all) JBTS-genes. Hh defects could explain many phenotypes associated with this disorder, whereby the retinal dystrophy may represent an exception. Indeed, retinal photoreceptors have such highly specialized cilia that cell-type specific roles of ciliary proteins are likely. Beyond that however, alterations in ciliary length, presence of cilia, or protein composition of the ciliary compartment may all result in aberrant Hh signaling, which in turn could explain patterning/differentiation/proliferation defects leading to signs such as polydactyly (patterning of the limb bud) or the MTS [for example, through deficient proliferation and premature differentiation of granule cell progenitors as shown in *talpid3* conditional knock-out mice (Bashford and Subramanian, 2019)]. Supporting the link between deficient Hh signaling and JBTS, is the fact that two of the more recently identified JBTS genes, namely *ARMC9* and *TOGARAM1*, were identified in a screen for Hh signaling. Zebrafish mutants in these genes display shortened cilia that have significantly decreased posttranslational tubulin modifications (acetylation and glutamylation), suggesting a stability defect for cilia lacking these proteins (Latour et al., 2020). This is associated with shorter cilia, which may affect signal transduction for morphological reasons. Moreover, tubulin posttranslational modifications are thought to alter protein interactions (e.g., motor proteins among others), which could also lead to defective Hh signaling component delivery (Breslow et al., 2018). While the ciliary localization of Armc9 and Togaram1 in zebrafish is still under investigation, work in other organisms has found these proteins, which function in a common module with JBTS proteins CSPP1 and CEP104, at the ciliary base and tip. Further work will be required to determine the role of JBTS-proteins in Hh signaling in zebrafish and the impact of their dysfunction in causing the observed phenotypes. The large number of zebrafish mutants now available in the community should allow to investigate this question conclusively.

One phenotype shared by virtually all zebrafish JBTS mutant models published to date is the body curvature in larvae and scoliosis in adults. The role of the Reissner fiber, a large aggregation of the glycoprotein SCO-spondin present in the central canal from head to tail tip, in maintaining a straight body axis has been clearly demonstrated (Vesque et al., 2019; Troutwine et al., 2020). Work on mutants in several ciliary genes has suggested a key role for motile ventricular cilia in the formation of this fiber (Cantaut-Belarif et al., 2018). Whether mutants in JBTS-genes display lack or immotility of these ventricular cilia, and whether the Reissner fiber is affected in these mutants remains open to investigation.

Another very consistent phenotype found in zebrafish JBTS models are the pronephric (kidney) cysts. Despite this frequently present feature, the pathomechanism leading from mutations in the respective JBTS gene to these cysts has been little investigated in JBTS zebrafish models. It may well be, that the motile cilia present in the zebrafish larval pronephric tubules play a different physiological role than the immotile primary cilia found on human renal tubular cells. In the zebrafish, it has been shown that lack of ciliary motility will cause kidney cysts (Kramer-Zucker et al., 2005; Sullivan-Brown et al., 2008; Zhao et al., 2013) (whereas cystic kidneys are not typically found in patients with ciliary motility dysfunction in primary ciliary dyskinesia). Moreover, the renal phenotype in individuals with JBTS consists not only of cysts but also of fibrosis, while fibrosis is unlikely to be present in the larval zebrafish pronephros, where the cysts are really formed by a very proximal dilatation of the tubules right after the glomerulus. So while the presence of pronephric cysts in zebrafish harboring mutations in JBTS genes appears to be a good indicator of ciliary dysfunction, it remains to be proven if the pathomechanism leading to kidney cysts is comparable between human and zebrafish larvae.

Finally, one major open question in the field of JBTS research, is the prominent variability observed between patients, in particular with respect to the non-CNS phenotypes. Indeed, even intra-familial variability is very frequently observed, suggesting the presence of genetic modifiers (Phelps et al., 2018). Whether these modifiers are present among JBTS genes themselves can now be tested thanks to the available zebrafish mutants, where double mutants or various combinations of heterozygous/homozygous alleles can be easily generated and studied. Moreover, the comparison between morphant and mutant phenotypes for a given gene can allow the identification of transcriptional adaptations, which can rescue or ameliorate a phenotype. Recent work based on the very mild *cep290* ^*fb208*^ mutant showed for example that *arl3*, *arl13b* and *unc119b*, which are important for cilia membrane transport, were upregulated in mutants but not in morphants to compensate for Cep290 loss (Cardenas-Rodriguez et al., 2021), thereby explaining the milder mutant than morphant phenotype. Another possible explanation for the observed phenotypic variability in individuals with JBTS is putative tissue-specific functions for JBTS proteins and/or cell-type specific protein isoforms. The variety of ciliated tissues in zebrafish allows to address the question of tissue-specificity by comparing ciliary phenotypes in different tissues of a given mutant. Indeed, among the JBTS mutants published to date, the severity of ciliary shortening appears to be variable in different tissues in the *togaram1* mutant for example (more severe in brain ventricles and less severe in olfactory placode cilia). Unfortunately, most studies so far have only focused on one given organ system such that a systematic analysis of all possible ciliopathy phenotypes is lacking (or has not been published) for most zebrafish JBTS mutant models, also limiting the conclusions that we can draw from the comparisons performed in this review. Future work based on comparisons of systematic analyses of the generated mutants could yield important insights, both into the question of tissue-specificity and to link dysfunction of the different subciliary compartments or protein modules to the distinct pathomechanisms.

## Future Directions

The long-term goal of disease modeling in general, including of ciliary research, is to improve clinical management and to develop therapeutic strategies for patients. The existing zebrafish mutant models for JBTS have helped to gain more insights into ciliary biology and JBTS pathomechanism. Unfortunately, the published phenotypic characterization of these models is largely incomplete. In the future, a systematic analysis of the generated mutants will certainly yield more insights into tissue-specific ciliary functions underlying JBTS pathomechanism. This knowledge should generate models to test, for example, linking ciliary localization of an affected protein to a specific pathomechanism in a given tissue or identifying which combinations of gene alleles modify the phenotype. Such models should in turn improve our ability to predict the clinical outcome for affected individuals.

One powerful advantage of the zebrafish model is its transparency at larval stages and the external development of the embryos. This enables live imaging of developing larvae in an intact whole-tissue context using transgenic lines in which the ciliary membrane, axoneme and/or basal body are fluorescently labeled. To fully understand the dynamic life of a cilium, it is key to observe it in its natural environment. So far, the few studies performing live imaging on cilia were mostly conducted on *in vitro* cell systems, in which ciliogenesis or ciliary resorption are artificially induced by removal or addition of serum (Ott and Lippincott-Schwartz, 2012; Ijaz and Ikegami, 2019; Mirvis et al., 2019). Using the zebrafish, these key events in the life of a cilium can be studied in a physiological context. Given the ease of transgenesis in zebrafish and combined with modern techniques such as optogenetics or chemogenetics, the mechanisms underlying other aspects of ciliary function, including signaling, can now be elucidated. As an example, recent work showed how cAMP is interpreted in the cilia of whole zebrafish embryos (Truong et al., 2021).

Another strength of the zebrafish model is the possibility of conducting large-scale chemical screens. Larvae can be placed in 96-well plates and exposed to chemical compounds in the medium. Many such high-throughput chemical screens have been performed using zebrafish (Cassar et al., 2020; Zhang and Peterson, 2020), including drug screenings in kidney disease (Gehrig et al., 2018) and retinal degeneration (Ganzen et al., 2021), yielding interesting candidate molecules. Given the large number of JBTS zebrafish models available, these can now be used for identifying compounds that improve ciliopathy phenotypes as a first step for developing therapeutic strategies for JBTS patients.

In conclusion, zebrafish JBTS models have already played an important role in shaping our understanding of ciliary biology and disease pathomechanism. A systematic assessment of the large number of zebrafish models generated by the community will allow to gain further insights into the role of JBTS proteins, to advance our knowledge on disease mechanisms and to identify therapeutic approaches, harnessing the strengths of this model system.
