# Supplementary material for: Insights Gained From Zebrafish Models for the Ciliopathy Joubert Syndrome
Source: Front Genet. 2022 Jun 30;13:939527. doi: 10.3389/fgene.2022.939527 (PMC9280682; doi:10.3389/fgene.2022.939527)
Supplement: Supplementary file 1 [file Table1.pdf]

JBTS gene	Model(s)	Kidney cilia defects	Floor plate cilia defects	Central canal cilia defects	KV cilia defects	Outer segment defects	Olfactory placode cilia defects	Neuromast defects	Ventricular cilia defects (larvae)	Ependymal cilia defects (adults)	References
ahi1[§]	MO	+	NA	NA	+	+	NA	NA	NA	NA	(Simms et al., 2012; Elsayed et al., 2015; Zhu et al., 2019)
	TALEN *iri46*, CRISPR	+	NA	NA	NA	+	NA	NA	NA	NA	(Lessieur et al., 2017; Zhu et al., 2019)
arl13b[§]	MO	+	NA	NA	+	NA	NA	NA	NA	NA	(Sun et al., 2004; Duldulao et al., 2009; Zhu et al., 2020)
	ENU *hi459*	+	NA	NA	NA	+	NA	NA	NA	NA	(Golling et al., 2002; Cantagrel et al., 2008; Duldulao et al., 2009; Song et al., 2016; Zhu et al., 2020)
armc9[§]	CRISPR *zh505*	+	NA	NA	NA	NA	+	NA	+	NA	(Latour et al., 2020)
b9d1	MO	-	NA	-	NA	NA	NA	NA	NA	NA	(Zhao and Malicki, 2011)
b9d2	MO	-	NA	-	NA	+	-	NA	NA	NA	(Dowdle et al., 2011; Zhao and Malicki, 2011)
cc2d2a[§]	ENU *w38**	-	-	NA	NA	+	-	-	NA	NA	(Gorden et al., 2008; Owens et al., 2008; Bachmann-Gagescu et al., 2011; Stawicki et al., 2016)
cep41	MO	+	NA	NA	NA	NA	+	NA	NA	NA	(J. E. Lee et al., 2012; Patowary et al., 2019; Ki et al., 2020)
	CRISPR *skk1*	NA	NA	NA	NA	NA	NA	NA	NA	NA	(Ki et al., 2020)
cep104	MO	-	NA	NA	+	NA	NA	NA	NA	NA	(Frikstad et al., 2019)
	CRISPR F0	NA	NA	NA	+	NA	NA	NA	NA	NA	(Frikstad et al., 2019)
cep120	MO	+	NA	+	NA	NA	NA	NA	NA	NA	(Shaheen et al., 2015)
cep290[§]	MO	-	NA	NA	+	+	-	NA	NA	NA	(Sayer et al., 2006; Schäfer et al., 2008; Baye et al., 2011; Murga-Zamalloa et al., 2011; Cardenas-Rodriguez et al., 2021)
	Tilling *fh297*, TALEN *fh378*, CRISPR *fb208**	-	NA	NA	-	+	-	-	NA	NA	(Stawicki et al., 2016; Lessieur et al., 2019; Cardenas-Rodriguez et al., 2021)
cspp1	MO	NA	-	NA	NA	-	NA	NA	NA	NA	(Tuz et al., 2014)
ift172[§]	MO	NA	NA	+	NA	+	+	NA	NA	NA	(Sun et al., 2004; Lunt et al., 2009; Halbritter et al., 2013; Bujakowska et al., 2015; Bergboer et al., 2018; Eisa-Beygi et al., 2018)
	Retroviral insertion *hi2211*	NA	NA	NA	NA	+	NA	NA	NA	NA	(Amsterdam et al., 1999; Sun et al., 2004; Gross et al., 2005; Lunt et al., 2009; Sukumaran and Perkins, 2009; Eisa-Beygi et al., 2018)
inpp5e[§]	MO	+	NA	NA	+	NA	NA	NA	NA	NA	(Luo et al., 2012; Xu et al., 2017)
	CRISPR	+	NA	NA	NA	NA	NA	NA	NA	NA	(Xu et al., 2017)
KIAA0556/katnip	MO	NA	NA	NA	NA	NA	NA	NA	NA	NA	(Roosing et al., 2016)
KIAA0568/talpid3[§]	ZFN *I262, I263, I264**	+	NA	+	+	+	NA	NA	NA	NA	(Ben et al., 2011; Ojeda Naharros et al., 2018)
KIAA0753/ofip	ENU *sa22657*	NA	NA	NA	NA	NA	NA	NA	NA	NA	(Hammarsjö et al., 2017)

JBTS gene	Model(s)	Kidney cilia defects	Floor plate cilia defects	Central canal cilia defects	KV cilia defects	Outer segment defects	Olfactory placode cilia defects	Neuromast defects	Ventricular cilia defects (larvae)	Ependymal cilia defects (adults)	References
kif7[§]	MO	NA	NA	NA	NA	NA	NA	NA	NA	NA	(Tay et al., 2005; Wilson et al., 2009; Putoux et al., 2011)
	ZFN *i271,i272** CRISPR *mw406*, CRISPR *co63*	NA	NA	+	NA	+	NA	NA	NA	NA	(Maurya et al., 2013; Lewis et al., 2017; Terhune et al., 2021)
mks1	MO	NA	NA	NA	NA	NA	NA	NA	NA	NA	(Leitch et al., 2008)
	CRISPR *w152*	NA	NA	NA	NA	NA	NA	-	NA	NA	(Stawicki et al., 2016)
nphp1	MO	-	NA	NA	-	NA	NA	NA	NA	NA	(Slanchev et al., 2011; Lindstrand et al., 2014)
ofd1	MO	NA	NA	NA	+	NA	NA	NA	NA	NA	(Ferrante et al., 2009; Lopes et al., 2011)
pde6d	MO	NA	NA	NA	NA	-	NA	NA	NA	NA	(Thomas et al., 2014)
rpgrip1l[§]	MO	NA	-	NA	NA	NA	NA	NA	NA	NA	(Khanna et al., 2009; Mahuzier et al., 2012)
	CRISPR F2	NA	-	+	NA	-	-	-	NA	+	(Vesque et al., 2019)
sufu	MO	NA	NA	NA	NA	NA	NA	NA	NA	NA	(Koudijs et al., 2005; Maurya et al., 2013)
tctn2	MO	NA	NA	NA	NA	NA	NA	NA	NA	NA	(Liu et al., 2018)
tmem67[§]	MO	NA	-	NA	NA	NA	-	-	NA	NA	(Adams et al., 2012; Leightner et al., 2013; Lee et al., 2017; Stayner et al., 2017)
	TALEN *e3*	+	NA	-	-	NA	NA	NA	NA	NA	(Zhu et al., 2021)
tmem138	MO	NA	NA	NA	NA	NA	NA	NA	NA	NA	(J. H. Lee et al., 2012)
tmem216[§]	MO	NA	NA	NA	NA	NA	NA	NA	NA	NA	(Valente et al., 2010; J. H. Lee et al., 2012)
	CRISPR *snyΔ175, snyR8Δ60*	NA	NA	NA	NA	+	NA	NA	NA	NA	(Liu et al., 2020)
tmem237	MO	NA	NA	NA	NA	NA	NA	NA	NA	NA	(Huang et al., 2011)
togaram1[§]	CRISPR *zh508, zh510*	+	NA	NA	NA	NA	+	NA	+	NA	(Latour et al., 2020)

REFERENCES

Adams, M., Simms, R. J., Abdelhamed, Z., Dawe, H. R., Szymanska, K., Logan, C. V., et al. (2012). A meckelin-filamin A interaction mediates ciliogenesis. *Hum Mol Genet* 21, 1272–1286. doi: 10.1093/hmg/ddr557

Amsterdam, A., Burgess, S., Golling, G., Chen, W., Sun, Z., Townsend, K., et al. (1999). A large-scale insertional mutagenesis screen in zebrafish. *Genes Dev* 13, 2713–2724. doi: 10.1101/gad.13.20.2713

Bachmann-Gagescu, R., Phelps, I. G., Stearns, G., Link, B. A., Brockerhoff, S. E., Moens, C. B., et al. (2011). The ciliopathy gene cc2d2a controls zebrafish photoreceptor outer segment development through a role in Rab8-dependent vesicle trafficking. *Hum Mol Genet* 20, 4041–4055. doi: 10.1093/hmg/ddr332

Baye, L. M., Patrinostro, X., Swaminathan, S., Beck, J. S., Zhang, Y., Stone, E. M., et al. (2011). The N-terminal region of centrosomal protein 290 (CEP290) restores vision in a zebrafish model of human blindness. *Hum Mol Genet* 20, 1467–1477. doi: 10.1093/hmg/ddr025

Ben, J., Elworthy, S., Ng, A. S. M., van Eeden, F., and Ingham, P. W. (2011). Targeted mutation of the talpid3 gene in zebrafish reveals its conserved requirement for ciliogenesis and Hedgehog signalling across the vertebrates. *Development* 138, 4969–4978. doi: 10.1242/dev.070862

Bergboer, J. G. M., Wyatt, C., Austin-Tse, C., Yaksi, E., and Drummond, I. A. (2018). Assaying sensory ciliopathies using calcium biosensor expression in zebrafish ciliated olfactory neurons. *Cilia* 7, 2. doi: 10.1186/s13630-018-0056-1

Bujakowska, K. M., Zhang, Q., Siemiatkowska, A. M., Liu, Q., Place, E., Falk, M. J., et al. (2015). Mutations in IFT172 cause isolated retinal degeneration and Bardet-Biedl syndrome. *Hum Mol Genet* 24, 230–242. doi: 10.1093/hmg/ddu441

Cantagrel, V., Silhavy, J. L., Bielas, S. L., Swistun, D., Marsh, S. E., Bertrand, J. Y., et al. (2008). Mutations in the cilia gene ARL13B lead to the classical form of Joubert syndrome. *Am J Hum Genet* 83, 170–179. doi: 10.1016/j.ajhg.2008.06.023

Cardenas-Rodriguez, M., Austin-Tse, C., Bergboer, J. G. M., Molinari, E., Sugano, Y., Bachmann-Gagescu, R., et al. (2021). Genetic compensation for cilia defects in cep290/NPHP6 mutants by upregulation of cilia-associated small GTPases. *J Cell Sci*. doi: 10.1242/jcs.258568

Dowdle, W. E., Robinson, J. F., Kneist, A., Sirerol-Piquer, M. S., Frints, S. G. M., Corbit, K. C., et al. (2011). Disruption of a ciliary B9 protein complex causes Meckel syndrome. *Am J Hum Genet* 89, 94–110. doi: 10.1016/j.ajhg.2011.06.003

Duldulao, N. A., Lee, S., and Sun, Z. (2009). Cilia localization is essential for in vivo functions of the Joubert syndrome protein Arl13b/Scorpion. *Development* 136, 4033–4042. doi: 10.1242/dev.036350

Eisa-Beygi, S., Benslimane, F. M., El-Rass, S., Prabhudesai, S., Abdelrasoul, M. K. A., Simpson, P. M., et al. (2018). Characterization of Endothelial Cilia Distribution During Cerebral-Vascular Development in Zebrafish (Danio rerio). *Arterioscler Thromb Vasc Biol* 38, 2806–2818. doi: 10.1161/ATVBAHA.118.311231

Elsayed, S. M., Phillips, J. B., Heller, R., Thoenes, M., Elsobky, E., Nürnberg, G., et al. (2015). Non-manifesting AHI1 truncations indicate localized loss-of-function tolerance in a severe Mendelian disease gene. *Hum Mol Genet* 24, 2594–2603. doi: 10.1093/hmg/ddv022

Ferrante, M. I., Romio, L., Castro, S., Collins, J. E., Goulding, D. A., Stemple, D. L., et al. (2009). Convergent extension movements and ciliary function are mediated by ofd1, a zebrafish orthologue of the human oral-facial-digital type 1 syndrome gene. *Hum Mol Genet* 18, 289–303. doi: 10.1093/hmg/ddn356

Frikstad, K.-A. M., Molinari, E., Thoresen, M., Ramsbottom, S. A., Hughes, F., Letteboer, S. J. F., et al. (2019). A CEP104-CSPP1 Complex Is Required for Formation of Primary Cilia Competent in Hedgehog Signaling. *Cell Rep* 28, 1907-1922.e6. doi: 10.1016/j.celrep.2019.07.025

Golling, G., Amsterdam, A., Sun, Z., Antonelli, M., Maldonado, E., Chen, W., et al. (2002). Insertional mutagenesis in zebrafish rapidly identifies genes essential for early vertebrate development. *Nat Genet* 31, 135–140. doi: 10.1038/ng896

Gorden, N. T., Arts, H. H., Parisi, M. A., Coene, K. L. M., Letteboer, S. J. F., van Beersum, Sylvia E C, et al. (2008). CC2D2A is mutated in Joubert syndrome and interacts with the ciliopathy-associated basal body protein CEP290. *Am J Hum Genet* 83, 559–571. doi: 10.1016/j.ajhg.2008.10.002

Gross, J. M., Perkins, B. D., Amsterdam, A., Egaña, A., Darland, T., Matsui, J. I., et al. (2005). Identification of zebrafish insertional mutants with defects in visual system development and function. *Genetics* 170, 245–261. doi: 10.1534/genetics.104.039727

Halbritter, J., Bizet, A. A., Schmidts, M., Porath, J. D., Braun, D. A., Gee, H. Y., et al. (2013). Defects in the IFT-B component IFT172 cause Jeune and Mainzer-Saldino syndromes in humans. *Am J Hum Genet* 93, 915–925. doi: 10.1016/j.ajhg.2013.09.012

Hammarsjö, A., Wang, Z., Vaz, R., Taylan, F., Sedghi, M., Girisha, K. M., et al. (2017). Novel KIAA0753 mutations extend the phenotype of skeletal ciliopathies. *Sci Rep* 7, 15585. doi: 10.1038/s41598-017-15442-1

Huang, L., Szymanska, K., Jensen, V. L., Janecke, A. R., Innes, A. M., Davis, E. E., et al. (2011). TMEM237 is mutated in individuals with a Joubert syndrome related disorder and expands the role of the TMEM family at the ciliary transition zone. *Am J Hum Genet* 89, 713–730. doi: 10.1016/j.ajhg.2011.11.005

Khanna, H., Davis, E. E., Murga-Zamalloa, C. A., Estrada-Cuzcano, A., Lopez, I., den Hollander, A. I., et al. (2009). A common allele in RPGRIP1L is a modifier of retinal degeneration in ciliopathies. *Nat Genet* 41, 739–745. doi: 10.1038/ng.366

Ki, S. M., Kim, J. H., Won, S. Y., Oh, S. J., Lee, I. Y., Bae, Y.-K., et al. (2020). CEP41-mediated ciliary tubulin glutamylation drives angiogenesis through AURKA-dependent deciliation. *EMBO Rep* 21, e48290. doi: 10.15252/embr.201948290

Koudijs, M. J., den Broeder, M. J., Keijser, A., Wienholds, E., Houwing, S., van Rooijen, Ellen M H C, et al. (2005). The zebrafish mutants dre, uki, and lep encode negative regulators of the hedgehog signaling pathway. *PLoS Genet* 1, e19. doi: 10.1371/journal.pgen.0010019

Latour, B. L., Van De Weghe, Julie C, Rusterholz, T. D., Letteboer, S. J., Gomez, A., Shaheen, R., et al. (2020). Dysfunction of the ciliary ARMC9/TOGARAM1 protein module causes Joubert syndrome. *J Clin Invest* 130, 4423–4439. doi: 10.1172/JCI131656

J. E. Lee, J. L. Silhavy, M. S. Zaki, J. Schroth, S. L. Bielas, S. E. Marsh, et al. (2012). CEP41 is mutated in Joubert syndrome and is required for tubulin glutamylation at the cilium. *Nat Genet* 44, 193–199. doi: 10.1038/ng.1078

J. H. Lee, J. L. Silhavy, J. E. Lee, L. Al-Gazali, S. Thomas, E. E. Davis, et al. (2012). Evolutionarily assembled cis-regulatory module at a human ciliopathy locus. *Science* 335, 966–969. doi: 10.1126/science.1213506

Lee, S.-H., Nam, T.-S., Li, W., Kim, J. H., Yoon, W., Choi, Y.-D., et al. (2017). Functional validation of novel MKS3/TMEM67 mutations in COACH syndrome. *Sci Rep* 7, 10222. doi: 10.1038/s41598-017-10652-z

Leightner, A. C., Hommerding, C. J., Peng, Y., Salisbury, J. L., Gainullin, V. G., Czarnecki, P. G., et al. (2013). The Meckel syndrome protein meckelin (TMEM67) is a key regulator of cilia function but is not required for tissue planar polarity. *Hum Mol Genet* 22, 2024–2040. doi: 10.1093/hmg/ddt054

Leitch, C. C., Zaghloul, N. A., Davis, E. E., Stoetzel, C., Diaz-Font, A., Rix, S., et al. (2008). Hypomorphic mutations in syndromic encephalocele genes are associated with Bardet-Biedl syndrome. *Nat Genet* 40, 443–448. doi: 10.1038/ng.97

Lessieur, E. M., Fogerty, J., Gaivin, R. J., Song, P., and Perkins, B. D. (2017). The Ciliopathy Gene ahi1 Is Required for Zebrafish Cone Photoreceptor Outer Segment Morphogenesis and Survival. *Invest Ophthalmol Vis Sci* 58, 448–460. doi: 10.1167/iovs.16-20326

Lessieur, E. M., Song, P., Nivar, G. C., Piccillo, E. M., Fogerty, J., Rozic, R., et al. (2019). Ciliary genes arl13b, ahi1 and cc2d2a differentially modify expression of visual acuity phenotypes but do not enhance retinal degeneration due to mutation of cep290 in zebrafish. *PLoS One* 14, e0213960. doi: 10.1371/journal.pone.0213960

Lewis, T. R., Kundinger, S. R., Pavlovich, A. L., Bostrom, J. R., Link, B. A., and Besharse, J. C. (2017). Cos2/Kif7 and Osm-3/Kif17 regulate onset of outer segment development in zebrafish photoreceptors through distinct mechanisms. *Dev Biol* 425, 176–190. doi: 10.1016/j.ydbio.2017.03.019

Lindstrand, A., Davis, E. E., Carvalho, C. M. B., Pehlivan, D., Willer, J. R., Tsai, I.-C., et al. (2014). Recurrent CNVs and SNVs at the NPHP1 locus contribute pathogenic alleles to Bardet-Biedl syndrome. *Am J Hum Genet* 94, 745–754. doi: 10.1016/j.ajhg.2014.03.017

Liu, C., Cao, R., Xu, Y., Li, T., Li, F., Chen, S., et al. (2018). Rare copy number variants analysis identifies novel candidate genes in heterotaxy syndrome patients with congenital heart defects. *Genome Med* 10, 40. doi: 10.1186/s13073-018-0549-y

Liu, Y., Cao, S., Yu, M., and Hu, H. (2020). TMEM216 Deletion Causes Mislocalization of Cone Opsin and Rhodopsin and Photoreceptor Degeneration in Zebrafish. *Invest Ophthalmol Vis Sci* 61, 24. doi: 10.1167/iovs.61.8.24

Lopes, C. A. M., Prosser, S. L., Romio, L., Hirst, R. A., O'Callaghan, C., Woolf, A. S., et al. (2011). Centriolar satellites are assembly points for proteins implicated in human ciliopathies, including oral-facial-digital syndrome 1. *J Cell Sci* 124, 600–612. doi: 10.1242/jcs.077156

Lunt, S. C., Haynes, T., and Perkins, B. D. (2009). Zebrafish ift57, ift88, and ift172 intraflagellar transport mutants disrupt cilia but do not affect hedgehog signaling. *Dev Dyn* 238, 1744–1759. doi: 10.1002/dvdy.21999

Luo, N., Lu, J., and Sun, Y. (2012). Evidence of a role of inositol polyphosphate 5-phosphatase INPP5E in cilia formation in zebrafish. *Vision Res* 75, 98–107. doi: 10.1016/j.visres.2012.09.011

Mahuzier, A., Gaudé, H.-M., Grampa, V., Anselme, I., Silbermann, F., Leroux-Berger, M., et al. (2012). Dishevelled stabilization by the ciliopathy protein Rpgrip1l is essential for planar cell polarity. *J Cell Biol* 198, 927–940. doi: 10.1083/jcb.201111009

Maurya, A. K., Ben, J., Zhao, Z., Lee, R. T. H., Niah, W., Ng, A. S. M., et al. (2013). Positive and negative regulation of Gli activity by Kif7 in the zebrafish embryo. *PLoS Genet* 9, e1003955. doi: 10.1371/journal.pgen.1003955

Murga-Zamalloa, C. A., Ghosh, A. K., Patil, S. B., Reed, N. A., Chan, L. S., Davuluri, S., et al. (2011). Accumulation of the Raf-1 kinase inhibitory protein (Rkip) is associated with Cep290-mediated photoreceptor degeneration in ciliopathies. *J Biol Chem* 286, 28276–28286. doi: 10.1074/jbc.M111.237560

Ojeda Naharros, I., Cristian, F. B., Zang, J., Gesemann, M., Ingham, P. W., Neuhauss, S. C. F., et al. (2018). The ciliopathy protein TALPID3/KIAA0586 acts upstream of Rab8 activation in zebrafish photoreceptor outer segment formation and maintenance. *Sci Rep* 8, 2211. doi: 10.1038/s41598-018-20489-9

Owens, K. N., Santos, F., Roberts, B., Linbo, T., Coffin, A. B., Knisely, A. J., et al. (2008). Identification of genetic and chemical modulators of zebrafish mechanosensory hair cell death. *PLoS Genet* 4, e1000020. doi: 10.1371/journal.pgen.1000020

Patowary, A., Won, S. Y., Oh, S. J., Nesbitt, R. R., Archer, M., Nickerson, D., et al. (2019). Family-based exome sequencing and case-control analysis implicate CEP41 as an ASD gene. *Transl Psychiatry* 9, 4. doi: 10.1038/s41398-018-0343-z

Putoux, A., Thomas, S., Coene, K. L. M., Davis, E. E., Alanay, Y., Ogur, G., et al. (2011). KIF7 mutations cause fetal hydrolethalus and acrocallosal syndromes. *Nat Genet* 43, 601–606. doi: 10.1038/ng.826

Roosing, S., Rosti, R. O., Rosti, B., Vrieze, E. de, Silhavy, J. L., van Wijk, E., et al. (2016). Identification of a homozygous nonsense mutation in KIAA0556 in a consanguineous family displaying Joubert syndrome. *Hum Genet* 135, 919–921. doi: 10.1007/s00439-016-1689-z

Sayer, J. A., Otto, E. A., O'Toole, J. F., Nurnberg, G., Kennedy, M. A., Becker, C., et al. (2006). The centrosomal protein nephrocystin-6 is mutated in Joubert syndrome and activates transcription factor ATF4. *Nat Genet* 38, 674–681. doi: 10.1038/ng1786

Schäfer, T., Pütz, M., Lienkamp, S., Ganner, A., Bergbreiter, A., Ramachandran, H., et al. (2008). Genetic and physical interaction between the NPHP5 and NPHP6 gene products. *Hum Mol Genet* 17, 3655–3662. doi: 10.1093/hmg/ddn260

Shaheen, R., Schmidts, M., Faqeih, E., Hashem, A., Lausch, E., Holder, I., et al. (2015). A founder CEP120 mutation in Jeune asphyxiating thoracic dystrophy expands the role of centriolar proteins in skeletal ciliopathies. *Hum Mol Genet* 24, 1410–1419. doi: 10.1093/hmg/ddu555

Simms, R. J., Hynes, A. M., Eley, L., Inglis, D., Chaudhry, B., Dawe, H. R., et al. (2012). Modelling a ciliopathy: Ahi1 knockdown in model systems reveals an essential role in brain, retinal, and renal development. *Cell Mol Life Sci* 69, 993–1009. doi: 10.1007/s00018-011-0826-z

Slanchev, K., Pütz, M., Schmitt, A., Kramer-Zucker, A., and Walz, G. (2011). Nephrocystin-4 is required for pronephric duct-dependent cloaca formation in zebrafish. *Hum Mol Genet* 20, 3119–3128. doi: 10.1093/hmg/ddr214

Song, P., Dudinsky, L., Fogerty, J., Gaivin, R., and Perkins, B. D. (2016). Arl13b Interacts With Vangl2 to Regulate Cilia and Photoreceptor Outer Segment Length in Zebrafish. *Invest Ophthalmol Vis Sci* 57, 4517–4526. doi: 10.1167/iovs.16-19898

Stawicki, T. M., Hernandez, L., Esterberg, R., Linbo, T., Owens, K. N., Shah, A. N., et al. (2016). Cilia-Associated Genes Play Differing Roles in Aminoglycoside-Induced Hair Cell Death in Zebrafish. *G3 (Bethesda)* 6, 2225–2235. doi: 10.1534/g3.116.030080

Stayner, C., Poole, C. A., McGlashan, S. R., Pilanthananond, M., Brauning, R., Markie, D., et al. (2017). An ovine hepatorenal fibrocystic model of a Meckel-like syndrome associated with dysmorphic primary cilia and TMEM67 mutations. *Sci Rep* 7, 1601. doi: 10.1038/s41598-017-01519-4

Sukumaran, S., and Perkins, B. D. (2009). Early defects in photoreceptor outer segment morphogenesis in zebrafish ift57, ift88 and ift172 Intraflagellar Transport mutants. *Vision Res* 49, 479–489. doi: 10.1016/j.visres.2008.12.009

Sun, Z., Amsterdam, A., Pazour, G. J., Cole, D. G., Miller, M. S., and Hopkins, N. (2004). A genetic screen in zebrafish identifies cilia genes as a principal cause of cystic kidney. *Development* 131, 4085–4093. doi: 10.1242/dev.01240

Tay, S. Y., Ingham, P. W., and Roy, S. (2005). A homologue of the Drosophila kinesin-like protein Costal2 regulates Hedgehog signal transduction in the vertebrate embryo. *Development* 132, 625–634. doi: 10.1242/dev.01606

Terhune, E. A., Cuevas, M. T., Monley, A. M., Wethey, C. I., Chen, X., Cattell, M. V., et al. (2021). Mutations in KIF7 implicated in idiopathic scoliosis in humans and axial curvatures in zebrafish. *Hum Mutat* 42, 392–407. doi: 10.1002/humu.24162

Thomas, S., Wright, K. J., Le Corre, S., Micalizzi, A., Romani, M., Abhyankar, A., et al. (2014). A homozygous PDE6D mutation in Joubert syndrome impairs targeting of farnesylated INPP5E protein to the primary cilium. *Hum Mutat* 35, 137–146. doi: 10.1002/humu.22470

Tuz, K., Bachmann-Gagescu, R., O'Day, D. R., Hua, K., Isabella, C. R., Phelps, I. G., et al. (2014). Mutations in CSPP1 cause primary cilia abnormalities and Joubert syndrome with or without Jeune asphyxiating thoracic dystrophy. *Am J Hum Genet* 94, 62–72. doi: 10.1016/j.ajhg.2013.11.019

Valente, E. M., Logan, C. V., Mougou-Zerelli, S., Lee, J. H., Silhavy, J. L., Brancati, F., et al. (2010). Mutations in TMEM216 perturb ciliogenesis and cause Joubert, Meckel and related syndromes. *Nat Genet* 42, 619–625. doi: 10.1038/ng.594

Vesque, C., Isabelle, A., Guillaume, P., Yasmine, C.-B., Alexis, E., Morgane, D., et al. (2019). Loss of the Reissner Fiber and increased URP neuropeptide signaling underlie scoliosis in a zebrafish ciliopathy mutant. *BioRxiv*.

Wilson, C. W., Nguyen, C. T., Chen, M.-H., Yang, J.-H., Gacayan, R., Huang, J., et al. (2009). Fused has evolved divergent roles in vertebrate Hedgehog signalling and motile ciliogenesis. *Nature* 459, 98–102. doi: 10.1038/nature07883

Xu, W., Jin, M., Hu, R., Wang, H., Zhang, F., Yuan, S., et al. (2017). The Joubert Syndrome Protein Inpp5e Controls Ciliogenesis by Regulating Phosphoinositides at the Apical Membrane. *J Am Soc Nephrol* 28, 118–129. doi: 10.1681/ASN.2015080906

Zhao, C., and Malicki, J. (2011). Nephrocystins and MKS proteins interact with IFT particle and facilitate transport of selected ciliary cargos. *EMBO J* 30, 2532–2544. doi: 10.1038/emboj.2011.165

Zhu, J., Wang, H.-T., Chen, Y.-R., Yan, L.-Y., Han, Y.-Y., Liu, L.-Y., et al. (2020). The Joubert Syndrome Gene arl13b is Critical for Early Cerebellar Development in Zebrafish. *Neurosci Bull* 36, 1023–1034. doi: 10.1007/s12264-020-00554-y

Zhu, L., Chen, L., Yan, L., Perkins, B. D., Li, S., Li, B., et al. (2019). Mutant Ahi1 Affects Retinal Axon Projection in Zebrafish via Toxic Gain of Function. *Front Cell Neurosci* 13, 81. doi: 10.3389/fncel.2019.00081

Zhu, P., Qiu, Q., Harris, P. C., Xu, X., and Lin, X. (2021). mtor Haploinsufficiency Ameliorates Renal Cysts and Cilia Abnormality in Adult Zebrafish tmem67 Mutants. *J Am Soc Nephrol* 32, 822–836. doi: 10.1681/ASN.2020070991
